# Positive psychotherapy and cognitive behavioral therapy in anxiety patients – A study protocol for a randomized control trial in an online group setting

**DOI:** 10.1371/journal.pone.0299803

**Published:** 2024-04-16

**Authors:** Catiana L. Engelhardt, Marina Meier, Sabrina Keller, Anton-Rupert Laireiter

**Affiliations:** Department of Psychology, Paris Lodron University of Salzburg, Salzburg, Austria; School of Nursing Sao Joao de Deus, Evora University, PORTUGAL

## Abstract

**Background:**

Anxiety disorders are common and debilitating which is why treatment is so important. According to the guidelines, Cognitive Behavioral Therapy (CBT) has the highest level of effectiveness among psychotherapeutic treatments and is the recommended procedure. However, not everyone responds well or at all to CBT which makes a wider range of therapy options valuable. Positive Psychotherapy (PPT) comes to mind as an alternative with its strength-based approach focusing on enhancing well-being and life satisfaction. Additionally, it has not yet been extensively studied how the processes that occur during treatment sessions and between treatment sessions effect treatment outcome. Thus, to lessen the lack of evidence regarding the efficacy of PPT as an anxiety treatment the planned study examines and compares the effectiveness of CBT and PPT as well as the effect of intrasession and intersession processes of the two therapy approaches.

**Method:**

The study is in the planning stage and consists of an efficacy and a process study. The efficacy study is a randomized controlled comparative study of patients with anxiety disorders (generalized anxiety disorder and/or panic disorder with or without agoraphobia) with two active treatment conditions (PPT and CBT) and a control group (CG; positive psychotherapy with minimal therapeutic supervision) in an online group setting. There are three measurement time points: before treatment begins (T0), at the end of the ten-week treatment (T1), and a follow-up after three months (T2). The aim of the study is to evaluate the efficacy of PPT and CBT in the treatment of anxiety disorders, and to compare the efficacy of online-based PPT with minimal therapeutic supervision and online-based PPT with intensive therapeutic supervision in the treatment of anxiety disorders. The process study will be used to evaluate both the intrasession processes and the intersession processes of the therapy in the two intervention groups. In addition, the process variables that predict the success of the therapy and the extent to which PPT and CBT differ in the therapy processes will be tested. The study is registered at the German Clinical Trial Register (№ DRKS00027521).

**Discussion:**

To our knowledge, this is the first randomized controlled comparative study to examine the effectiveness of CBT and PPT for anxiety disorders in an online group setting.

## Introduction

### Anxiety disorders—Epidemiology and treatment approaches

Anxiety disorders are more widespread and represent one of the most common mental disorders [1, 2]. According to international studies, the lifetime prevalence ranges between 14% and 29% [2, 3], whereby the quality of life of those affected is often severely restricted. With regard to the so-called “Years Lived with Disability” (YLD), i.e. the years of life lived with disability, according to the World Health Organization (WHO) in 2015 anxiety disorders are in sixth place worldwide. They are thus primarily chronic diseases [4]. Anxiety disorders are associated with a high utilization of the health care system [5] and extremely comorbid with other mental illnesses [6, 7], such as depression and alcohol dependence [8]. According to the S3 guidelines for the treatment of anxiety disorders, psychotherapy and pharmacotherapy ought to be offered equally. After giving thorough information about the various treatment options, the patient’s preference should be considered. For all anxiety disorders, CBT has the highest level of evidence and recommendation of all psychotherapeutic methods. In the case of insufficient effectiveness of one form of therapy, the other or a combination of both should be offered [5, 9]. Psychodynamic psychotherapy has been included in the current S3 guidelines, with the recommendation that this form of psychotherapy be offered when CBT has proved ineffective, is not available or when there is a preference for it on the part of the informed patient [5].

### Positive psychotherapy

PPT is a science-based psychotherapy that enhances positive emotions, character strengths, and meaning-making in a direct way, with the goal of reversing psychopathologies and promoting the experience of happiness [10]. In contrast to traditional deficit-oriented treatment models, PPT strives to focus on human well-being and satisfaction. The central assumption is that the evocation and promotion of positive emotions, the awareness of one’s own strengths and the sense of meaning are particularly efficient in the treatment of mental disorders, as people are subject to the so-called negativity bias, especially in stressful life situations. This negativity bias describes the phenomenon that negative thoughts, feelings and experiences have a greater psychological impact than neutral or positive ones [11]. PPT strives to compensate for this bias by focusing attention on positive things. According to [12], a lack of positive emotions and well-being can not only be a symptom but also a cause of mental disorders.

### Previous efficacy studies on positive psychotherapy

Before PPT was evaluated as an integrated treatment concept, individual interventions were empirically validated [13]. Individual Positive Psychology interventions have been shown to improve psychological well-being, promote hope and enjoyment, advance psychological rehabilitation and self-esteem, and positively influence psychopathological symptoms [13, 14]. In a meta-analysis of the effectiveness of positive psychology interventions, [15] found small to moderate effects on well-being, strengths, quality of life, depression, anxiety, and stress. Numerous studies regarding the effectiveness of positive-psychological interventions conducted on the disorder depression demonstrated a significant reduction in symptoms and an increase in well-being [13]. [14] found that individual PPT with severely depressed patients resulted in symptom improvement and longer remissions compared to usual care including medication therapy. Several studies have demonstrated moderate to strong effects in moderate to severe disorder severity, as well as remission rates of up to one year compared to waiting list control groups or patients treated with antidepressants and psychiatric monitoring [12, 16–18]. For depression disorder PPT is also highly effective when compared to CBT, as shown in the randomized-controlled comparative study by [19]. Their results revealed large effects for the reduction of depressive symptomatology, while only small to medium effect sizes were found for CBT. PPT was also superior to CBT in terms of positive outcomes such as life satisfaction and subjective well-being. Additionally, PPT lead to significant effects in the treatment of borderline patients [20] as well as in the treatment of nicotine dependence [21]. In summary, based on the studies conducted to date, it can be assumed that PPT is an effective method for several different psychological disorders.

### Positive psychotherapy for anxiety disorders

Empirical studies on the effectiveness of PPT for anxiety disorders are rare. In studies on the effectiveness of Well-Being Therapy (WBT) by [22], which combines both co-behavioural methods and those of Positive Psychology, significant results were achieved. In a controlled comparative study, CBT was compared with WBT in patients with Generalized Anxiety Disorder in a group setting. The study was able to confirm the superiority of WBT both after the end of therapy and in the follow-up examination after one year [23]. However, due to the small sample size (*N* = 9), the significance of this study must be considered low. Another study by Goodwin [Unpublished] was able to show a reduction in anxiety after a ten-week intervention in the form of a PPT treatment. [24] used a clinical sample (*N* = 29) to examine the efficacy of combined PPI intervention on anxiety symptomatology over a treatment period of ten weeks. The study resulted in participants having significantly lower anxiety symptoms and a significantly higher well-being in the pre-post comparison in the intervention group compared to those in the waiting list control group. These effects were still observed in the follow-up (6 months). Like the PPT according to [10], the intervention by [24] was conducted in a manualized manner and strongly resembles the PPT manual in terms of the structure of the units and contains comparable interventions.

### Intrasession and intersession processes

According to [25, 26], the process research phase in psychotherapy represents the fourth and current phase of psychotherapy research. It was preceded by the legitimation phase, the competition phase and the prescription phase. Process research is concerned with investigating the mechanisms of action of psychotherapeutic treatments. Intrasession processes refer to all those changes that occur directly in response to specific events in the therapy session. Whereas intersession processes are defined as conscious processes of change between two sessions that are brought about by representations of the session [27]. [28] showed that approximately 90% of all patients engage with therapy or their therapist between sessions. Patients who are more intensely involved in the therapeutic process also show more intense representations [29, 30] and a better therapy outcome [31]. It has also been shown that patients’ positive perceptions of therapy progress are associated with greater involvement in intersession process thoughts and actions [32]. A clear correlation between positive representations and a good therapeutic relationship has been found [33–36].

### Effectiveness of video-assisted therapy

The importance of virtual care has grown strongly especially due to the Covid-19-Pandemic. Additionally, video-assisted therapy is becoming increasingly important and has been used more frequently. In a large-scale meta-analysis, online interventions from 108 randomized controlled trials for 25 different clinical impairments were examined from the period 2000 to 2012 [37]. Consequently, the effectiveness of online therapy for depression, social phobia, and panic disorders can be considered empirically proven. Further studies are available for eating disorders [38], pathological gambling [39], complicated grief [40], substance-related addictions [41] and post-traumatic stress disorders [42], anxiety [43] and functional disorders [37], among others. The results of internet-based interventions are promising. For depression, reported effect sizes from five meta-analyses range from *d* = 0.41 to *d* = 0.94 [44], and online programs for anxiety disorders have an average symptom reduction of *d* = 0.92 [45]. These results show that online treatments lead to the same neuronal changes as those observed after successful classical psychotherapy [46]. Another meta-analysis on the effectiveness of online psychotherapy, which included 92 studies with a total of 9764 participants, showed that the mean treatment effect (Cohen’s *d*) between pre- and postmeasurement was 0.53, averaged across all disorders and forms of intervention. This effect size is quite comparable to that of conventional outpatient therapy [47]. [48] found that both video-based therapy and traditional therapy achieved particularly good effects. Therapeutic success with video-assisted therapy is strongest when using the CBT treatment modality and for anxiety, depression, and Post-Traumatic Stress Disorder. [49] conducted a study of adolescents with anxiety disorders. While one group received a face-to-face intervention, the other group received video-assisted therapy with less therapeutic support. Both groups showed a significant decrease in anxiety symptomatology, even when compared to a waitlist control group. These effects persisted over time (12-month follow-up). Computer-assisted CBT for anxiety showed similar clinical outcomes to face-to-face psychotherapy [50, 51].

### Hypotheses and research questions

The main research question of the efficacy study is: Does treatment with PPT and CBT lead to a) a reduction in anxiety symptomatology and to a b) an improvement in general life satisfaction and to c) the promotion of happiness compared to treatment with PPT with minimal therapeutic support after completion of treatment (T1) as well as three months after the treatment has ended (T2). Previous data suggest that Positive Psychology also has exceptionally good effects on anxiety disorders (Goodwin [Unpublished], [24]). Another important question concerns the evaluation of the intrasession processes and the intersession processes of the two intervention groups. The aim is to examine which process variables predict the success of therapy and whether there are differences between the forms of therapy.

## Methods

### Design and setting

**Fig 1:**
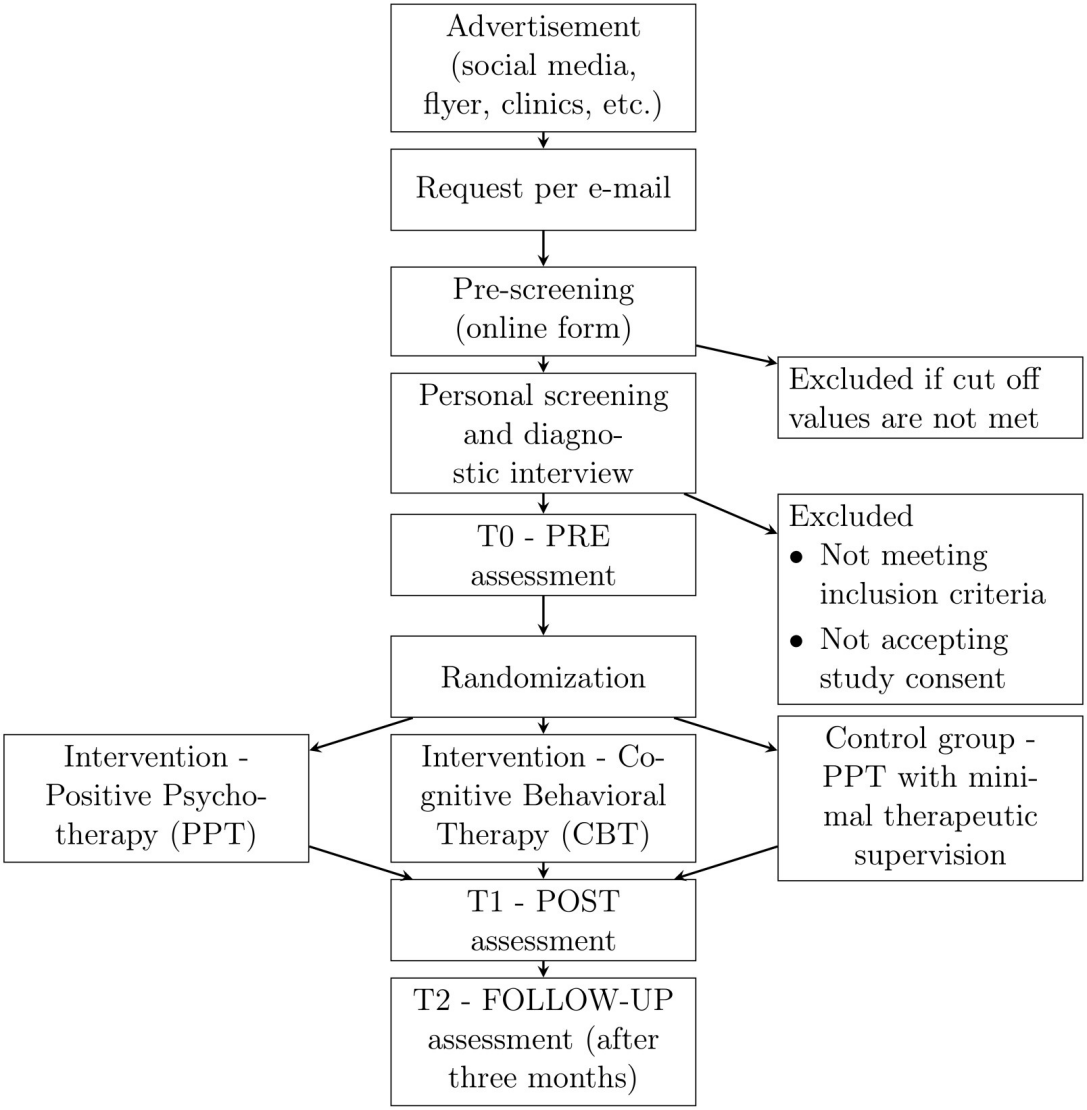
Flow diagram of the study design.

The planned study is a randomized-controlled comparative study and will be realized as a ten-week online group therapy with the following three treatment conditions to which participants will be randomly assigned: Group 1: An intervention condition in which participants receive PPT treatment, Group 2: an intervention condition in which they receive CBT treatment, and Group 3: a control condition designed as PPT treatment with minimal therapeutic supervision. Participants in the control condition receive the same material, content, and exercises as the participants in PPT treatment condition. However, they only have three online video call meetings and work through the content on their own during the remaining weeks. There are three measurement time points for testing the efficacy hypotheses: An online questionnaire survey before treatment begins (PRE measure), a second identical survey after treatment will be completed (POST measure), and a third identical survey three months after treatment will be ended (FOLLOW-UP measure). This results in a 3×3 between- and within-subjects design. The hypotheses regarding the intra- and intersession processes will be tested by means of weekly surveys in each case before the therapy sessions (intersession processes) as well as after the therapy sessions (intrasession processes). Fig 1 shows the study design and the different steps of the procedure in a chronic flow diagram. The study is conducted at the Department of Clinical Psychology and Psychotherapy at the University of Salzburg. Prior to initiation, the study design was submitted to and approved by the Ethics Committee of the University of Salzburg (EK-GZ: 22/2021). The study is registered at the German Clinical Trial Register (№ DRKS00027521; date of registration February, 3^rd^ 2022; type of registration: prospective). The full details according to the World Health Organization Trial Registration Data Set standards are shown in Table 1. For a complete description of the relevant information and their position in the manuscript according to the SPIRIT checklist, please refer to S1 Checklist.

**Table 1:** Items from the World Health Organization Trial Registration Data Set.

Data Category	Information
Primary registry and trial identifying number	German Clinical Trials Register № DRKS00027521
Date of registration in primary registry	February, 3^rd^ 2022
Secondary identifying numbers	N/A
Source(s) of monetary or material support	Financial contributions from the Paris Lodron University of Salzburg, Austria
Primary sponsor	Stiftungs- und Förderungsgesellschaft der Paris Lodron Universität Salzburg, Austria
Secondary sponsor	N/A
Contact for public queries	Paris Lodron Universität Salzburg, Austria, Catiana Engelhardt, phone: +49 1604039525, e-mail: s1076028@stud.sbg.ac.at
Contact for scientific queries	Univ.-Prof. Dr. Anton-Rupert Laireiter, Paris Lodron Universität Salzburg, Austria
Public title	Positive Psychotherapy and Cognitive Behavioral Therapy in Anxiety Patients
Scientific title	Positive Psychotherapy and Cognitive Behavioral Therapy in Anxiety Patients—A randomized controlled Trial with three Conditions in an online Group Setting
Countries of recruitment	Austria, Germany
Health conditions or problems studied	ICD10: F41.1 Generalized anxiety disorder, F41.0 Panic disorder [episodic paroxysmal anxiety], F40.1 Social phobias
Intervention(s)	Active comparator 1: Positive psychotherapy, Active comparator 2: Cognitive behavioral therapy, Control group: online positive psychotherapy with minimal therapeutic supervision
Key inclusion and exclusion criteria	Ages eligible for study: between 18 and 65 years, Sexes eligible for study: both, Inclusion: sufficient intellectual capacity and knowledge of the German language, presence of one of the following disorder diagnoses according to F41.0 panic disorder with and without agoraphobia and/or, F41.1 generalized anxiety disorder and/or, F40.1 social phobia, Exclusion: concurrent or planned participation in psychotherapeutic or psychological treatment or counseling or participation in other psychological group services in the following three months, presence of one or more of the following disorders: major depressive episode, bipolar affective disorder or mania (current as well as history), schizophrenic or schizoaffective disorder, grief reaction, severe anorexia or bulimia, substance dependence (alcohol, illicit drugs), severe Personality Disorder; acute suicidality; use of psychotropic drugs itself not a criterion for exclusion, but a change in medication, a change in dose, or complete discontinuation of the drug in the past or next three months
Study type	Interventional, randomized control study, masking: none, primary purpose: comparative therapy quality assessment
Date of first enrollment	Planned: March, 1^st^ 2022
Target sample size	165
Recruitment status	Recruiting
Primary outcomes	Extent of anxiety, subjective experience of happiness, quality of life
Key secondary outcome	General psychological distress

### Feasibility

The feasibility of the study design was examined as part of a master’s thesis by one of the study assistants. For this purpose, the first study round was evaluated for the effectiveness and feasibility of the interventions, as well as the satisfaction with the therapy from the perspective of the participants and therapists. The results of the work showed high feasibility of the study design, high satisfaction with the therapy, and trends indicating effectiveness of the treatment for anxiety disorders. The detailed results of the feasibility study can be found in the master’s thesis of [52].

### Participants

This trial will include patients who meet the following criteria. Inclusion criteria:

- age between 18 and 65 years
- sufficient intellectual capacity and knowledge of the German language
- presence of one of the following disorder diagnoses according to ICD-10
  - F41.0 panic disorder with and without agoraphobia and/or
  - F41.1 generalized anxiety disorder and/or
  - F40.1 social phobia

Exclusion criteria:

- concurrent or planned participation in psychotherapeutic or psychological treatment or counseling or participation in other psychological group services in the following three months
- presence of one or more of the following disorders: major depressive episode, bipolar affective disorder or mania (current as well as history), schizophrenic or schizoaffective disorder, grief reaction, severe anorexia or bulimia, substance dependence (alcohol, illicit drugs), severe Personality Disorder
- acute suicidality
- use of psychotropic drugs itself not a criterion for exclusion, but a change in medication, a change in dose, or complete discontinuation of the drug in the past or next three months

### Sample size

The sample size was calculated using a power analysis (G*Power 3.1; [53]), given an alpha error of.05 and a power ≥.80, resulting in a sample size of *N* = 165. Accordingly, 55 persons would be included in each of the three intervention groups. Effect size estimates resulted from findings of previous studies [12, 19]. Since a high dropout rate is to be expected, a significantly higher number of subjects will be screened and admitted to the study.

### Participant recruitment process

Recruitment will be conducted using a variety of strategies. Part of the recruitment will be done by distributing flyers in pharmacies, doctors’ and psychotherapists’ offices, and other public buildings such as universities, churches, supermarkets, and schools. In addition, clinics, physicians, and psychotherapists will be contacted directly by mail and asked to make patients aware of our offer. At the same time, the study will be advertised online via various social media channels. On the one hand, Facebook is used to post in disorder-specific groups and make those who are affected aware of our offer directly. Instagram is the second platform we will use to promote the study. To do this, an Instagram page will be created for the study. Through this page, various influencers related to the study topic will be contacted via direct message and asked to use their reach and share the study. In addition, own topic-related posts will be created and shared, and advertisements will be placed for the posts. Interested people can contact us via mail to the study mail address, which was communicated via the various advertising tools. To check the general suitability for the study and to filter out suitable participants, interested people will undergo a pre-screening form, which was carried out in the form of an online questionnaire. Based on the evaluation of the pre-screening questionnaire and previously determined cut-off values, a screening appointment will be arranged with suitable applicants. Unsuitable applicants will receive a rejection by e-mail which includes contact details for psychotherapeutic support services. The screening will take place via an online video call using the provider Zoom. It will be conducted by psychologists and master psychology students employed within the study project. In the screening, the German version of the short form of the Diagnostic Interview in Psychological Disorders (MINI-DIPS; [54]) and the German version of the Personality Disorder Screening—Short Form (PSS-K; [55]) will be used to check inclusion as well as exclusion criteria and to formulate a diagnosis. In addition, information about the procedures and contents of the study and the various data collection procedures will be given, and informed consent will be obtained from the participants to participate in the study and to complete the mandatory evaluation questionnaires. All participants will be also given the telephone number of the crisis service responsible for their area so that they can contact them in the event of an emergency.

### Randomization and blinding

With the help of the online platform randomizer.org participants will be randomly allocated to three test conditions right after they completed the PRE-evaluation form. The allocation is random and independent of the diagnosis or other criteria. The externally recruited therapists will be blindly assigned to the treatment conditions. Neither the participants nor the practitioners were allowed to decide which group they were assigned to. The authors, who also act as therapists, will conduct the control group to prevent falsification effects in the intervention groups. Statistical analyses will be conducted by the principal investigator, who will not be blinded or independent.

### Interventions

The first step in conducting the study was the development of the treatment manuals and therapy materials. Two treatment manuals were developed, one each for PPT and CBT treatment. The PPT manual is based on the official PPT manual by [10] and adapted for the study. Because of economic reasons instead of 15 sessions as in the original manual, only 10 therapy sessions are conducted in the present study. In addition, the individual PPT topics were adapted and exercises from the personal PPT application repertoire of the study leaders were incorporated. As in the original manual, a gratitude diary will be filled out daily by the participants accompanying the therapy. In the individual sessions, various positive-psychological interventions are carried out, e.g., participants learn how to handle and use positive emotions, work out their personal character strengths and learn how to use them in concrete anxiety situations. Other topics covered in the PPT treatment include gratitude, optimism, a positive self-image, self-compassion, resilience, everest goals, best-possible self, and future perspective. The CBT manual is conceptually adapted to the PPT treatment and also comprises 10 therapy sessions. The exercises and content were selected based on the German language therapy tools for anxiety disorders by [56]. In the CBT treatment, participants are required to practice the relaxation exercise Progressive Muscle Relaxation according to Jacobsen on a daily basis. In CBT treatment, participants learn how to deal with anxiety thoughts and behavior and how to restructure stressful anxiety related cognitions. For this purpose, various cognitive-behavioral therapy interventions are carried out, e.g., the development of an individual explanatory model, the worry chair and various attention and breathing exercises. An elementary part of the treatment is an exposure exercise, which is worked towards during therapy. Other topics covered in CBT treatment include the three levels of anxiety (body, thoughts, behavior), the vicious cycle of anxiety, and mindfulness. In addition to the treatment manuals, workbooks were created for the participants to work on during the therapy. The workbooks contain all organizational details, theoretical topics as well as all exercises and worksheets that must be done in the video-supported sessions or as homework. Additional websites (created by Webflow) were provided for both intervention groups to make additional therapy tools available, such as videos or questionnaires. That aside the websites are also used to conduct part of the data collection. The implementation of the treatments takes place online via videoconferencing using the Zoom platform. Participants in the two intervention conditions (PPT & CBT) will meet once a week with the treatment providers for a 120-minute online presence meeting, in which the content of the respective sessions will be conducted according to the manual. In addition, participants will be given homework assignments to complete independently for the week between the face-to-face sessions. These homework assignments will be discussed in the group at the beginning of each session. The control group is designed as a PPT with minimal therapeutic supervision. The participants will receive the same PPT workbook as the intervention group and accordingly will work on the same topics and exercises over the ten weeks as the participants in the PPT intervention group. The difference between the two groups lies in the therapeutic support. In the control group, a video-supported meeting with the therapist will be held only three times: In the first session, halfway through therapy in the fifth session, and in the last session. In between, participants receive an overview email at the beginning of the week and a reminder email at the end of the week. Apart from the possibility of mail contact with the therapists, the participants in the control group carry out the exercises independently with the help of the workbook and the materials on the website. For the control group, as for the intervention groups, a separate website is provided. Another important component of all treatment conditions is the formation of teams of two clients (couple teams) within each therapy group. These teams will be formed in the first session of therapy and will remain in place throughout the duration of therapy. Participants are encouraged to meet at least once a week to discuss the week’s exercises and topics with each other. In some cases, specific reflection questions will be provided in the individual exercises in the workbook. Depending on the possibilities of the participants, the meeting could take place online via a telephone call or video call or in person. When forming the couple teams, deliberate care was taken to divide the participants according to local proximity.

### Outcome measures

Proposed psychological outcome measures are as follows. All named questionnaires are based on self-report.

#### Primary outcome measures

- extent of anxiety as measured by self-report questionnaires
  - Beck Anxiety Inventory German version (BAI; [57])
  - Panic and Agoraphobia Scale German version (PAS; [58])
  - Generalized Anxiety Disorder 7 German version (GAD-7; [59])
  - Fear Questionnaire German version (FQ; [60])
- subjective experience of happiness
  - Positive Psychotherapy Inventory German version (PPTI; [10])
  - Flourishing Scale German version (FS; [61])
- quality of life
  - Satisfaction with Life Scale German version (SWLS; [62])

For additional third-party assessment of the level of anxiety a questionnaire designed by the authors was used (the following topics were queried: tension, anxious mood, fear, sleep disturbance, depressiveness, anxious behavior, physical symptomatology, psychological distress, impairment in living, life satisfaction, motivation & energy).

#### Secondary outcome measures

- General psychological distress
  - ICD-10 symptom rating German version (ISR [63])
  - Patient Health Questionnaire German version (PHQ 9 [64])

For the evaluation of intra- & intersession processes intra-session processes will be assessed using the Patient Session-Assessment Questionnaire German version (STU-P, ger. orig.: “Stunden Beurteilungsbogen für Patienten”; [65]) and intersession processes will be assessed using the Intersession-Questionnaire German version (ISF, ger. orig.: “Intersession Fragebogen”; [66]).

### Data collection

**Fig 2:**
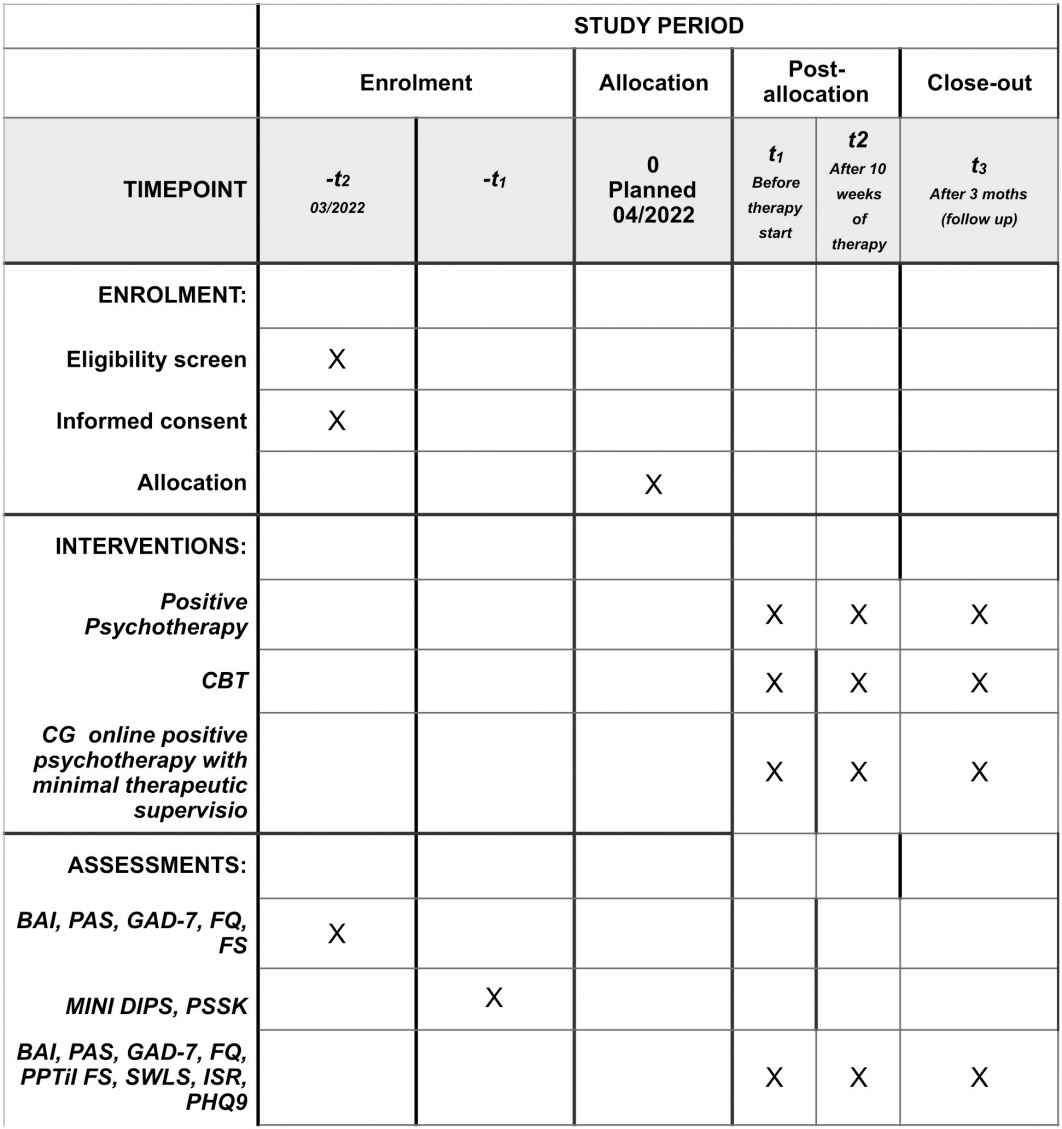
SPIRIT schedule of enrollment.

All self-report measures will be collected by computerized questionnaires, using different online survey platforms. For T0, T1 & T2 evaluation (see Fig 2), Google forms will be used. For evaluating intra- and intersession processes forms included at our study websites will be used. Table 2 shows an overview of all measurement instruments used across the different measurement time points of the study. Access to any data is restricted to trial personnel and investigators. Name, e-mail address and telephone number are required for organizational purposes and for sending the links for the online data collection. These data are recorded independently of the other study-related data only by the study director in an Excel spreadsheet and kept in a password-protected laptop. Nobody else has access to it. All other data will be collected pseudonymously via a subject code to be generated by the patients themselves (= individual code). Anonymity is to be protected on different levels. At the level of the practitioner, it is protected by the professional laws. The practitioners are bound to secrecy according to the Austrian Psychologists’ Act and the German Psychotherapists’ Act. Accordingly, no information about the persons and their treatment may be disclosed to the outside. Only during supervision and intervision may the contents of the units be discussed anonymously. However, the supervisors are also subject to the same professional laws as the therapists, so that the data of the subjects are also protected at this level. At the level of research data, the pseudonymization by means of individual codes makes it impossible to assign the data to any specific person, unless the person discloses his or her individual code. At the level of the actual personal data, anonymity is ensured by the fact that only the study director has access to this data and only is aware of it. However, even he or she cannot establish an assignment of the names to the data because he or she does not have the individual codes. The personal data are accessible only through a password-protected table in an equally protected laptop.

**Table 2:** Diagnostic materials.

Assessments	Study period						
	Prescreening	Screening	PRE evaluation (T0) Before session	Intervention (10 weeks) After session		POST evaluation (T1)	FOLLOW-UP evaluation (T2, after 3 months)
				Before session	After session		
BAI	×		×			×	×
PAS	×		×			×	×
GAD-7	×		×			×	×
FQ	×		×			×	×
PPTI			×			×	×
FS			×			×	×
SWLS			×			×	×
ISR			×			×	×
PHQ-9			×			×	×
STU-P					×		×
ISF				×			
MINI-DIPS		×					
PSS-K		×					
Informed consent		×					
Third-party evaluation by therapists		After screening	Before therapy			After therapy	

BAI: Beck Anxiety Inventar, PAS: Panic and Agoraphobia Scale, GAD-7: Generalized Anxiety Diagnostic, FQ: Fear Questionnaire, PPTI: Positive Psychotherapy Inventory, FS: Flourishing Scale, SWLS: Satisfaction with Life Scale, ISR: ICD-10 Symptom Rating, PHQ-9: Patient Health Questionnaire, STU-P Patient Session-Assessment Questionnaire (ger. orig.: “Stunden Beurteilungsbogen für Patienten”), ISF: Intersession-Questionnaire (ger. orig.: “Intersession Fragebogen”), MINI-DIPS: Diagnostic short interview for mental disorders (ger. orig.: “Diagnostisches Kurzinterview bei psychischen Störungen”), PSS-K: Personality Disorder Screening—Short Form (ger. orig.: “Persönlichkeitsstörung-Screening—Kurzform”)

### Statistical analysis

The primary outcome is pre-post and follow-up change in anxiety and positive outcomes (overall life satisfaction, happiness experience). In this study, we will conduct an intention-to-treat analysis. For the statistical analysis of the primary outcome, we will conduct a repeated measures ANOVA with three independent variables (PPT, CBT, CG). For the process study, two ANOVAs will be conducted with two independent variables each (emotional experience positive/negative and level of session activity high/low).

### Trial governance

The Trial Management Group consists of the principal investigator, the study director (A.- R. Laireiter) and is supported by two study assistants. The Management Group will provide overall management of the study including set-up of the study, recruitment, managing mails, and interpretation of results. In addition, eleven therapists (psychological psychotherapists in training) support the study by conducting the therapy groups. In preparation for the study, therapy manuals, websites and workbooks were developed for the different intervention groups by the principal investigator, the study director and a study assistant. Overall supervision of the trial is provided by the principal investigator. The therapists were trained in the manuals and for screenings by the project management. All therapists were trained in both manuals in order to be able to adopt both intervention groups (PPT and CBT) as needed during the course of the various study runs. At regular intervals, the therapy sessions are checked for their manual compliance and the therapists receive supervision by qualified psychotherapists and supervisors.

## Discussion

This study investigates the efficacy of Positive Psychotherapy and Cognitive Behavioral Therapy in patients with anxiety disorders in an online-based group setting. Conventional treatments for anxiety disorders focus primarily on cognitive strategies and relaxation techniques, as well as exposure [5]. The treatment focus of Positive Psychotherapy is on positive affect and enhancing general well-being. So far, research focused on the efficacy of (online-based) CBT as a treatment for anxiety and is referred to as the gold standard for treating a wide range of disorders. Current studies show that Positive Psychology Interventions prove to be effective for depression and psychological well-being [16, 67] as well as anxiety [68, 69]. However, until now no study concentrated on comparing the efficacy of both treatments (CBT & PPT) in people struggling with anxiety disorders in an online-based group setting. Thus, no concrete evidence has been found which supports using PPT to treat anxiety disorders or other mental disorders. Positive Psychotherapy may be a much-needed alternative for people not responding well to CBT and the online-group setting makes the therapy more accessible to people with restricted mobility or severe symptoms. Since people with anxiety often lead lives in which they avoid situations or places that trigger their fears and become more withdrawn as a result, online interventions could facilitate entry into therapy. Another potential strength is that online (group) therapy can be considered a cost-effective setting [70, 71]. Times like the coronavirus pandemic, where therapy sessions had to be postponed or other ways to continue had to be figured out, display the usefulness of online therapy. Additionally, it is unclear which process variables predict therapy success. The impact of intersession and intrasession processes for therapy success has not been extensively studied. However, recent studies suggest a positive correlation between these processes and a positive therapy result [32, 72]. In this study we want to explore whether intrasession and/or intersession processes contribute to the effectiveness of the treatment and if so, whether a difference can be observed depending on the treatment (CBT or PPT). In the present study, a pure waiting list control group design is deliberately not used, since this allows statements on the absolute effectiveness of a treatment, but is weak in terms of the effectiveness of treatments in comparison to already established and comparable approaches. In addition, wait-list control groups are widely used in research, whereas comparisons with active alternative treatments are less common.

### Limitations

Due to the online design of this study, it is more difficult to motivate and regulate the subjects’ participation. Therefore, we heavily relied on the intrinsic motivation (in addition to the overall satisfaction rate regarding the treatment) and commitment of the participants, which was met with considerably great interindividual variability. Since only individuals from mainly Germany and Austria were recruited, general statements about the results of our study can only be drawn for the western population. For future research, a more global-based study should be considered. Nonetheless, we expect that our study reveals valuable results that further research regarding online-based group therapy and contribute to a better understanding of the possible variables affecting therapy success. If proven that positive psychotherapy is beneficial or even as effective as cognitive behavioral therapy in patients with anxiety disorders, this could provide an alternative to conventional therapy approaches.

## Supporting information

S1 Checklist SPIRIT checklist. (PDF)
